# Effects of advanced glycation end products (AGEs) on the differentiation potential of primary stem cells: a systematic review

**DOI:** 10.1186/s13287-023-03324-5

**Published:** 2023-04-11

**Authors:** Kuishuai Xu, Liang Zhang, Ning Yu, Zhongkai Ren, Tianrui Wang, Yingze Zhang, Xia Zhao, Tengbo Yu

**Affiliations:** 1 Department of Sports Medicine, The Affiliated Hospital of Qingdao University, Qingdao, 266000 Shandong China; 2 Department of Abdominal Ultrasound, The Affiliated Hospital of Qingdao University, Qingdao, 266000 Shandong China; 3 Department of Traumatology, The Affiliated Hospital of Qingdao University, Qingdao, 266000 Shandong China

**Keywords:** Advanced glycation end products, Stem cells, Differentiation, Review

## Abstract

The formation and accumulation of advanced glycation end products (AGEs) have been associated with aging and the development, or worsening, of many degenerative diseases, such as atherosclerosis, chronic kidney disease, and diabetes. AGEs can accumulate in a variety of cells and tissues, and organs in the body, which in turn induces oxidative stress and inflammatory responses and adversely affects human health. In addition, under abnormal pathological conditions, AGEs create conditions that are not conducive to stem cell differentiation. Moreover, an accumulation of AGEs can affect the differentiation of stem cells. This, in turn, leads to impaired tissue repair and further aggravation of diabetic complications. Therefore, this systematic review clearly outlines the effects of AGEs on cell differentiation of various types of primary isolated stem cells and summarizes the possible regulatory mechanisms and interventions. Our study is expected to reveal the mechanism of tissue damage caused by the diabetic microenvironment from a cellular and molecular point of view and provide new ideas for treating complications caused by diabetes.

## Introduction

With the development of regenerative medicine, stem cell transplant-based replacement therapy has become an important treatment approach [1]. Stem cells are a cell population with self-renewal capacity and multilineage differentiation potential that can differentiate into different types of cells under specific conditions [2]. For example, bone marrow stem cells (BMSCs) can differentiate into osteoblasts, adipocytes, chondrocytes, etc., after different stimuli [3–5]. Periodontal ligament stem cells (PDLSCs) migrate and differentiate into osteoblasts to repair alveolar bone defects after periodontal tissue injury for repair [6]. Neural stem cells (NSCs) can be isolated from rodents' embryonic tissues and human brain tissues and can differentiate into various cell lineages, including neurons, astrocytes, and oligodendrocytes. At present, exogenous NSCs transplantation has been applied to treat neurological diseases, including vascular dementia [7], traumatic brain injury [8], spinal cord injury [9], and stroke [10]. Adipose tissue-derived stem cells (ADSCs) are widely sourced and have the effects of restoring tissue cells and promoting cell regeneration [11]. Tendon stem cells (TSDCs), found in the Achilles tendon or tendon, have multilineage differentiation potential and can reverse tendinopathy and promote osteotendinous junction healing [12]. Although stem cell transplantation has a good tissue repair ability for injured tissues, local harmful microenvironments such as oxidative stress and inflammatory stimuli result in low stem cell survival. Among them, AGEs can be deposited in various parts and organs of the body under abnormal pathological conditions such as diabetes and aging and form a microenvironment that is not conducive to stem cell differentiation in the body. At the same time, the deposition of AGEs leads to corresponding tissue damage and dysfunction of repair function, causing serious damage to body tissues and organs.

AGEs are polymers produced by non-enzymatic reactions between proteins, lipids, nucleic acids, and glucose, formed in three steps: (1) Schiff base is produced by non-enzymatic saccharification of the aldehyde group of early reducing sugars with proteins; (2) Schiff base forms more stable Amadori products through structural rearrangement; (3) Amadori products undergo further structural changes after dehydration and degradation, finally forming AGEs. AGEs are structurally diverse compounds divided into endogenous AGEs produced in vivo and exogenous AGEs ingested in vitro [13]. The way collagen forms AGEs can also be divided into intermolecular cross-linking modification and side chain modification. Partially cross-linked structures give rise to autofluorescence signatures, whereas side-chain modification forms AGEs that typically do not have autofluorescence signatures. In addition to in vivo synthesis, numerous studies have highlighted that exogenous AGEs, especially dietary AGEs intake, can significantly affect the levels of AGEs in the body [14]. In addition to dietary sources, AGEs can also be found in cigarettes. Roasted tobacco leaves have been suggested as a source of substances that promote increased AGEs in vivo. Although exogenous and endogenous AGEs are thought to have different sources, recent observations suggest that they may act synergistically to cause AGEs to produce greater harm [15].

AGEs were first thought to be easily formed in tissues with a slow metabolism, and their increased content is closely related to aging. AGEs have a role in the development of diseases such as kidney [16], retina [17], cardiovascular disease [18], and osteoporosis [19]. Also, AGEs have a stronger toxic effect on pancreatic β-cells than high glucose and a longer duration of action. AGEs are involved in diabetes, and diabetes-related complications mainly occur through the following aspects: (1) AGEs and protein cross-linking change the biological characteristics of proteins [20], which causes changes in physiological and biochemical properties and leads to functional damage to the body, such as vascular thickening, reduced elasticity, and vascular endothelial dysfunction. (2) AGEs interact with their receptor of advanced glycation end products (RAGE) to activate a series of complex signal transduction pathways and induce many intracellular signal transduction pathways to produce reactive oxygen species and reactive nitrogen species, which further lead to different pathological responses [21]. (3) AGEs promote pancreatic β-cell apoptosis by inducing the production of reactive oxygen species and increasing the expression of RAGE, while they can significantly reduce apoptosis by reducing the production of reactive oxygen species and inhibiting RAGE [22]. Overall, AGEs accumulate rapidly during hyperglycemia and oxidative stress and are important factors involved in the development of diabetes and the continuous deterioration of its complications [23]. Yet, the effect of AGEs on primary stem cell differentiation is still controversial, and effective strategies to reverse the adverse effects of AGEs on stem cell differentiation are currently lacking.

This systematic review summarizes the effects of AGEs on the cell differentiation potential of different types of primary isolated stem cells and elaborates and summarizes the relevant mechanisms and interventions of AGEs on the differentiation potential of stem cells. These data may improve the theoretical basis for revealing the hazards of AGEs and promoting the application of stem cell therapy.

## Data and methods

### Source of data

PubMed and Web of Science electronic databases were searched for relevant articles published from inception of the database to November 6, 2022. Articles on the effects of AGEs on primary stem cell differentiation were identified by identifying PICO elements (P = population: primary stem/progenitor cells, I = intervention: AGEs, C = comparison: control, O = results: differentiation). In addition, the authors used MeSH to find English-written and published articles on advanced glycation end products and stem cells using the same keywords as used in recent literature [1].

### Literature screening criteria

Exclusion criteria were: (1) the studied cells were not stem cells or progenitor cells; (2) the study involved only diabetes or high glucose, not AGEs; (3) a review study, case report, book, announcement, meeting, etc.; (4) the study results were not related to stem cell differentiation; (5) the study was not a cell experiment performed at the level of stem cells; (6) non-English literature.

### Data extraction and literature quality evaluation

Selected articles were screened and assessed by two reviewers (KSX and LZ) according to exclusion criteria and were excluded when both reviewers considered the article to be ineligible. Finally, data were grouped according to stem cell type: bone marrow stem cells (Table 1), periodontal ligament stem cells (Table 2), adipose tissue-derived stem cells (Table 3), neural stem cells (Table 4), tendon stem cells (Table 5), CD34 progenitor cells (Table 6), and endothelial progenitor cells (Table 7).

**Table 1:** Summary of included studies using BMSCs isolated from the bone marrow

Study name	Year	Isolation SC	AGEs application		Effect on outcome			Regulation mechanism	Intervention factors
			Concentration	Duration	Osteogenesis	Chondrogenesis	Adipogenesis		
Kim et al.[24]	2013	Rat bone marrow	300 μg/ml	1d	↓	–	–	Ang1/Tie2 pathway	COMP-Ang1
Guo et al.[25]	2021	Rat bone marrow	50, 100, 200 μg/mL	1, 2, 3d	↓	–	↑	Sirt3	rAAV-Sirt3/CCCP
Stolzing et al.[26]	2010	Rat bone marrow	10, 50, 200, 300, 500, 700, 100 mol/μl	14d	↓	–	–	–	**–**
Kume et al.[27]	2005	Human bone marrow	10, 100 μg/mL	21d	↓	↓	↓	–	**–**
Notsu et al.[28]	2014	Human bone marrow	200 μg/ml	7, 14, 21d	↓	–	–	TGF-β	SD208
Larsen et al.[29]	2012	Human bone marrow	0.75 mM, 1 mM	3d	↓	–	–	–	–
Wang et al.[30]	2021	Rat bone marrow	200 μg/ml	7d, 14d	↓	–	–	–	Adrenomedullin 2
Waqas et al.[31]	2022	Human bone marrow	400, 600, 800, 1000 μM	1d, 4d, 11d, 20d	↓	–	–	RAGE	**–**
Okazaki et al.[32]	2012	Mouse bone marrow	10, 50 100, 200 μg/ml	3d, 7d, 14d, 21d	↓	–	–	Osterix expression	**–**

Summary of isolation procedures and sampling, AGEs concentration, duration of application, effects on stem cell differentiation, mechanisms of regulation and pre-measures

BMSCs: Bone marrow stem cells; AGEs: Advanced glycation end products; SC: stem cells; ↓: decrease; ↑: increase

**Table 2:** Summary of studies that included PDLSCs

Study name	Year	Isolation SC	AGEs application	Effect on outcome	Effect on outcome		Regulation mechanism	Intervention factors
			Concentration	Duration	Osteogenesis	Adipogenesis		
Liu et al.[33]	2015	Human periodontal ligament	1, 10, 100, 200 ng/mL	3, 7d	↓	↓	Wnt/β‑catenin pathway	DKK1
Wang et al.[34]	2019	Human periodontal ligament	200 μg/ml	14, 28d	↓	**–**	PKCβ2 phosphorylation	GLP-1
Zhang et al.[35]	2019	Human periodontal ligament	200 µg/ml	5, 7, 14, 21d	↓	**–**	Wnt/β‑catenin pathway	Berberine
Guo et al.[36]	2019	Human periodontal ligament	50, 100, 200 μg/mL	14d	↓	**–**	–	**–**
Wang et al.[37]	2022	Human periodontal ligament	25, 50 100, 150, 200 μg/ml	24 h, 48 h, 72 h	↓	–	RAGE	Periostin

Summary of isolation procedures and sampling, AGEs concentration, duration of application, effects on stem cell differentiation, mechanisms of regulation and pre-measures

PDLSCs: Periodontal ligament stem cells; AGEs: Advanced glycation end products; SC: stem cells; ↓: decrease; ↑: increase

**Table 3:** Summary of studies that included ADSCs

Study name	Year	Isolation SC	AGEs application		Effect on outcome			Regulation mechanism	Intervention factors
			Concentration	Duration	Osteogenesis	Endothelial cells	Adipogenesis		
Zhang et al.[38]	2018	Rats Fat	40, 80, 120, 160 μg/mL	1, 4, 7d	↓	–	–	Wnt/β-catenin pathway	FPS-ZM1
Li et al.[39]	2020	Mouse Fat	20, 40, 80, 160 μg/mL	1, 2, 4d	↓	–	–	Wnt/β-catenin pathway	–
Guo et al.[40]	2017	Human Fat	100 mg/L	4, 8, 16d	–	↓	–	–	–
Xiao et al.[41]	2020	Human Fat	10 mg/mL	3, 7, 14d	↓	–	↑	miR-1248/CITED2/HIF-1α pathway	MiRNA-1248
Li et al.[42]	2022	Rats Fat	20, 40, 80 μg/mL	24, 48, 96 h	↓	–	–	SIRT3	Irisin

Summary of isolation procedures and sampling, AGEs concentration, duration of application, effects on stem cell differentiation, mechanisms of regulation and pre-measures

ADSCs: Adipose tissue-derived stem cells; AGEs: Advanced glycation end products; SC: stem cells; ↓: decrease; ↑: increase

**Table 4:** Summary of studies that included NSCs

Study name	Year	Isolation SC	AGEs application		Effect on outcome		Regulation mechanism	Intervention factors
			Concentration	Duration	Neuronal differentiation	Astrocytic differentiation		
Wang et al.[43]	2009	Rat-brain tissue samples	200, 400 mg/L	3, 7d	↓	–	–	–
Bao et al.[44]	2020	Mouse-brain tissue samples	100 μg/mL	7d	↓	–	HDAC3	–
Guo et al.[45]	2013	Rat-brain tissue samples	400 μg/mL	7d	↓	↑	Notch-Hes1 pathway	–

Summary of isolation procedures and sampling, AGEs concentration, duration of application, effects on stem cell differentiation, mechanisms of regulation and pre-measures

NSCs:Neural stem cells; AGEs: Advanced glycation end products; SC: stem cells; ↓: decrease; ↑: increase

**Table 5:** Summary of studies that included TDSCs

Study name	Year	Isolation SC	AGEs application		Effect on outcome		Regulation mechanism	Intervention factors
			Concentration	Duration	Osteogenesis	Others		
Xu et al.[46]	2019	Rat Achilles tendon	100, 200, 400 μg/ml	5d	↑	–	–	Pioglitazone

Summary of isolation procedures and sampling, AGEs concentration, duration of application, effects on stem cell differentiation, mechanisms of regulation and pre-measures

TDSCs: Tendon stem cells; AGEs: Advanced glycation end products; SC: stem cells; ↓: decrease; ↑: increase

**Table 6:** Summary of included studies using CD34 progenitor cells isolated from blood

Study name	Year	Isolation SC	AGEs application		Effect on outcome		Regulation mechanism	Intervention factors
			Concentration	Duration	Vasculogenesis	Others		
Scheubel et al.[47]	2006	Human Blood	2, 20, 200 mg/ml	3d	↓	–	–	**–**

Summary of isolation procedures and sampling, AGEs concentration, duration of application, effects on stem cell differentiation, mechanisms of regulation and pre-measures

AGEs: Advanced glycation end products; SC: stem cells; ↓: decrease; ↑: increase

**Table 7:** Summary of included studies using EPCs isolated from the bone marrow

Study name	Year	Isolation SC	AGEs application		Effect on outcome		Regulation mechanism	Intervention factors
			Concentration	Duration	Osteogenesis	Others		
Wang et al.[48]	2022	Rat bone marrow	10, 20 40, 80, 100 μg/ml	5 min, 15 min, 30 min, 7d	↑	–	MAPK pathway	**–**

Summary of isolation procedures and sampling, AGEs concentration, duration of application, effects on stem cell differentiation, mechanisms of regulation and pre-measures

EPCs: Endothelial progenitor cells; AGEs: Advanced glycation end products; SC: stem cells; ↓: decrease; ↑: increase

### Study selection

Through database searches of PubMed and Web of Science, 244 and 343 articles were found, respectively. A total of 212 duplicate articles were removed, resulting in 375 articles. Based on the exclusion criteria for literature screening, 350 articles were excluded after the screening, and 25 studies were finally included in this review.

## Effect of AGEs on the differentiation of different types of primary stem cells

### Bone marrow stem cells

Nine articles [24–32] investigated the effects of AGEs on differentiation from BMSCs (Table 1) derived from the bone marrow of rats [24–26, 30], humans [31, 37–39], or mice [32]. The ability of AGEs to inhibit the osteogenic differentiation of BMSCs was observed in all nine studies [24–32]. In addition, one [27] study found that AGEs inhibit the chondrogenic differentiation ability of BMSCs, while another reported opposing results. Guo et al*.* [25] found that the adipogenic differentiation ability of rat bone marrow-derived BMSCs was enhanced after the application of AGEs, while Kume et al*.* [27] found that the adipogenic differentiation ability of human bone marrow-derived BMSCs was reduced after the application of AGEs. In short, AGEs inhibit both osteogenic and chondrogenic differentiation of BMSCs; however, there is still controversy regarding their adipogenic differentiation.

### Periodontal ligament stem cells

Five articles have investigated the effects of AGEs on the differentiation of mesenchymal stem cells from the periodontal ligament (Table 2). All stem cells were derived from the human periodontal ligament, and all studies [33–37] suggested that AGEs have an inhibitory effect on the osteogenic differentiation of PDLSCs. Moreover, Liu et al*.* showed that AGEs down-regulates the adipogenic differentiation potential of PDLSCs [33].

### Adipose tissue-derived stem cells

Five studies reported the effects of AGEs on the differentiation of ADSCs (Table 3). ADSCs were collected from subcutaneous fat in the groin of rats [38, 42], mice [39], or humans [40, 41]. Four studies [38, 39, 41, 42] reported that AGEs suppress the osteogenic potential of ADSCs under osteoinductive conditions in a dose-dependent manner, significantly reducing ALP activity and decreasing the expression of osteoblast-specific genes. Furthermore, Guo et al*.* [40] reported that AGEs led to a decrease in the differentiation potential of ADSCs into endothelial cells, while Xiao et al*.* [41] found that AGEs promote adipogenesis in ADSCs.

### Neural stem cells

NSCs were reported in three articles (Table 4). Cultures of proliferating neurospheres were obtained from rat [43, 45] or mouse [44] brain tissue. Wang et al*.* [43] and Bao et al*.* [44] found that AGE-BSA inhibits the formation of neurospheres and neuronal differentiation in an approximately concentration-dependent manner. Guo et al*.* [45] conducted a more in-depth study based on Wang’s results and found that AGEs promote astrocyte differentiation while inhibiting neuronal formation. However, this study had limitations related to experimental design, so it needs to be further validated by including more time points and concentration gradients.

### Tendon stem cells

One study reported the effect of AGEs on the differentiation of TDSCs (Table 5) derived from rat tendons. Xu et al*.* [46] applied AGEs to TDSCs for 5 days. ALP and alizarin red staining showed that AGEs promote the differentiation of TDSCs toward osteogenesis. Yet, so far, no data have been reported on the effects of AGEs on osteogenic marker genes in tendon stem cells, and the investigators did not further study the potential mechanism of AGE-induced ossification of TDSCs. Accordingly, more work is needed in the future to elaborate related mechanisms.

### CD34 progenitor cells

Scheubel et al*.* [47] showed that AGEs decrease the angiogenic potential of CD34 progenitor cells derived from human blood (Table 6).

### Endothelial progenitor cells

One study reported the effect of AGEs on the differentiation of EPCs derived from rat bone marrow (Table 7). Wang et al*.* [48] showed that AGEs might bind to RAGE on the membrane of endothelial cells, thereby leading to an increase in differentiation toward osteogenesis.

## Potential mechanisms of AGEs affecting primary stem cell differentiation

Previous studies have explored and explained the potential reasons AGEs affect primary stem cell differentiation; however, few studies on pathways exist. The most investigated and relatively well-established mechanisms mainly include AGE/RAGE [24, 37, 38], the Wnt/β-catenin pathway [33, 35, 38, 39], and the Notch-Hes1 pathway [45].

### Mechanisms of AGEs affecting BMSCs differentiation

Kim et al*.* [24] found that AGEs down-regulate the phosphorylation of AKT and p38 through the Ang1/Tie2 signaling pathway and induce diminished osteogenic differentiation ability of BMSCs. Angiopoietin 1 (Ang1) is a ligand for the Tie2 receptor [49]. Many studies related to diabetes have shown that the Ang1/Tie2 signaling system has a key role in vascular growth and maturation [50]. Previous studies have also confirmed Ang1 as a factor regulating apoptosis in MSCs [51].

Waqas et al*.* [31] suggested that the interaction of AGEs with RAGE is one reason for the decreased osteogenic potential of BMSCs. Okazaki et al*.* [32] found that the mechanism through which AGEs inhibit osteogenic differentiation of BMSCs may be related to decreased osteocalcin expression and increased RAGE expression. Furthermore, Notsu et al*.* [28] found that the increase in transforming growth factor-beta (TGF-β) by AGEs through binding to RAGE may be one of the reasons affecting stem cell differentiation ability. TGF-β is a multifunctional polypeptide with a regulatory role in injured tissue repair, embryonic development, bone tissue regeneration, and stem cell proliferation and differentiation [52]. TGF-β is highly expressed in ribs, spinal cartilage, and perichondrium and is abundant in the bone matrix, which can bind to β3-specific receptors on cell membranes and affect cell division and proliferation and the synthesis of extracellular matrix [53]. Meanwhile, TGF-β has an important role in osteogenesis and is one of the important regulators [54], and TGF-β can bind to the promoters of Runx2 and OCN, which in turn affect the expression of osteogenic genes [55, 56].

TGF-β3 is a subtype of TGF-β, and its research in tissue wound repair, cartilage healing, scar repair, and fibrous tissue formation is relatively mature [57, 58]. In recent years, with the continuous upgrading of biological scaffold materials, the effect of TGF-β3 on promoting and inducing the proliferation and osteogenic and chondrogenic differentiation of adult stem cells derived from biological scaffold materials [59], especially in the early stage of osteogenesis [60], has been extensively studied. Deng et al*.* [61] found that TGF-β3 could induce osteogenic differentiation of human BMSCs, thereby stimulating bone regeneration. Li et al*.* [62] demonstrated that TGF-β3 promotes osteogenic differentiation of PDLSCs by activating MAPK channels. In summary, TGF-β is a key factor in regulating osteogenesis, which has an important role in stem cell differentiation.

There are seven members of the mammalian Sirtuins family (including Sirt1-7). Sirt3 is located in the mitochondria and is a major component of mitochondrial deacetylases, which affect most of the key aspects of mitochondrial homeostasis [63–67]. Guo et al*.* [25] found that Sirtuin 3-mediated mitotic phagocytosis regulates the AGEs-induced osteogenic differentiation potential of BMSCs.

Osteogenic differentiation is an energy-consuming process in which the biosynthesis and oxidative energy supply of mitochondria are greatly increased, and the amount of ROS, its metabolic by-products, also correspondingly increases. Therefore, maintaining homeostasis of mitochondrial function and biosynthesis is essential for osteogenic differentiation [68]. Unfortunately, the decrease in the osteogenic differentiation potential of BMSCs induced by AGEs through Sirt3 has not been more intensively studied for the downstream targets of Sirt3, and the potential signaling pathways also need further study exploration.

### Mechanisms of AGEs affecting PDLSCs differentiation

**Fig. 1:**
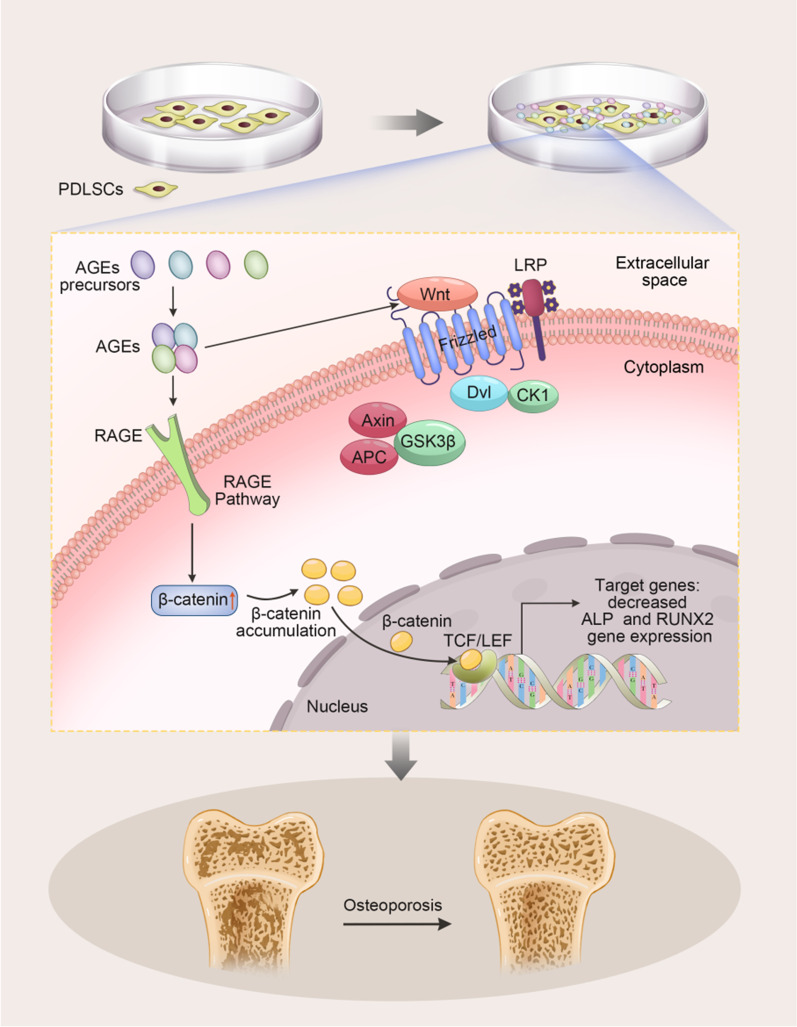
AGEs attenuate the osteogenic differentiation ability of PDLSCs by activating canonical Wnt/β-catenin signaling (Liu et al., 2015; Zhang et al., 2019). In PDLSCs, Wnt ligands interact with Frizzled to activate the Wnt signaling pathway, whereas AGEs further activate the Wnt/β-catenin signaling pathway, which leads to increased expression of phosphorylated β-catenin. As a result, β-catenin translocates into the nucleus, binds to TCF/LEF, and induces decreased expression of ALP and RUNX2

AGEs influence the osteogenic differentiation potential of PDLSCs through the Wnt signaling pathway. Liu et al*.* [33] and Zhang et al. [35] found that AGEs reduce the osteogenic differentiation ability of PDLSCs by activating the canonical Wnt/β-catenin pathway. Wnt/β-catenin or canonical Wnt is a signaling pathway that has an important regulatory role in stem cell self-renewal and differentiation. In PDLSCs, Wnt ligands interact with Frizzled to activate the Wnt signaling pathway, whereas AGEs further activate the Wnt/β-catenin signaling pathway, which leads to increased expression of phosphorylated β-catenin. As a result, β-catenin translocates into the nucleus, binds to TCF/LEF, and induces decreased expression of ALP and RUNX2** (**Fig. 1**).**

Some previous studies have shown that activation of the Wnt/β-catenin pathway can promote osteogenic differentiation of BMSCs, ADSCs, and PDLSCs [69–72], and the neuronal differentiation process of neural stem cells is also regulated by the Wnt/β-catenin signaling pathway [73, 74]. Wnt is a family of 19 secreted glycoproteins that mediate developmental processes by regulating cell proliferation, differentiation, and apoptosis [75]. GSK-3β is inhibited when the canonical Wnt/β-catenin signaling pathway is activated. Then, β-catenin accumulates, translocates to the nucleus, and binds to T cell factor/lymphoid enhancer-binding factor transcription factors, leading to the transcription of Wnt downstream target genes [76]. Wnt proteins transduce a variety of signaling cascades, including the canonical Wnt/β-catenin pathway, the Wnt/ca2^+^ pathway, and the Wnt/polarity pathway [77]. Previous studies have confirmed that canonical Wnt/β-catenin signaling has a huge role in maintaining bone homeostasis and significantly increases alkaline phosphatase (ALP) activity [78, 79]. However, the role of the Wnt signaling pathway on osteoblast differentiation remains controversial, and more studies have shown that the Wnt signaling pathway inhibits osteoblast differentiation [80–82]. Wnt signaling has also been studied in detail in abnormal neuronal differentiation of neural stem cells, and inhibition of the Wnt signaling pathway has a significant inhibitory role in the in vitro differentiation of NSCs (83). The discovery of the Wnt signaling pathway may preliminarily reveal the effect of AGEs on the abnormal differentiation of primary stem cells and provide theoretical and experimental clues for rescuing the abnormal differentiation status of AGEs on primary stem cells, but its potential molecular mechanism still needs to be further explored.

Wang et al*.* [34] found that AGEs affect the osteogenic potential of PDLSCs through PKCβ2; during this process, the expression of RAGE is up-regulated, PKCβ2 activity is increased, and the ability of osteogenic differentiation is decreased. Osteogenic gene and protein expression showed corresponding up- and down-regulation after adding PKC inhibitor (LY333531) and activator (PMA), respectively. Protein kinase C (PKC) is a serine/threonine protein kinase with important physiological functions in many intracellular signaling pathways. Hyperactivation of PKCβ2 isoforms is particularly closely related to the occurrence and development of diabetic cardiovascular complications. PKCβ2 has an important role in the development of diabetic complications, and membrane displacement and phosphorylation are important markers of PKC activation [84, 85]. Overactivation of PKCβ2 promotes increased reactive oxygen species (ROS) production, which causes tissue damage in the body [86–88]. AGEs can act on RAGE and activate PKC, leading to the release of superoxide, which has an important role in periodontal diseases [89, 90]. However, the underlying molecular mechanism through which PKCβ2 phosphorylation impacts the differentiation of PHLSCs requires further investigation.

### Mechanisms of AGEs affecting ADSCs differentiation

**Fig. 2:**
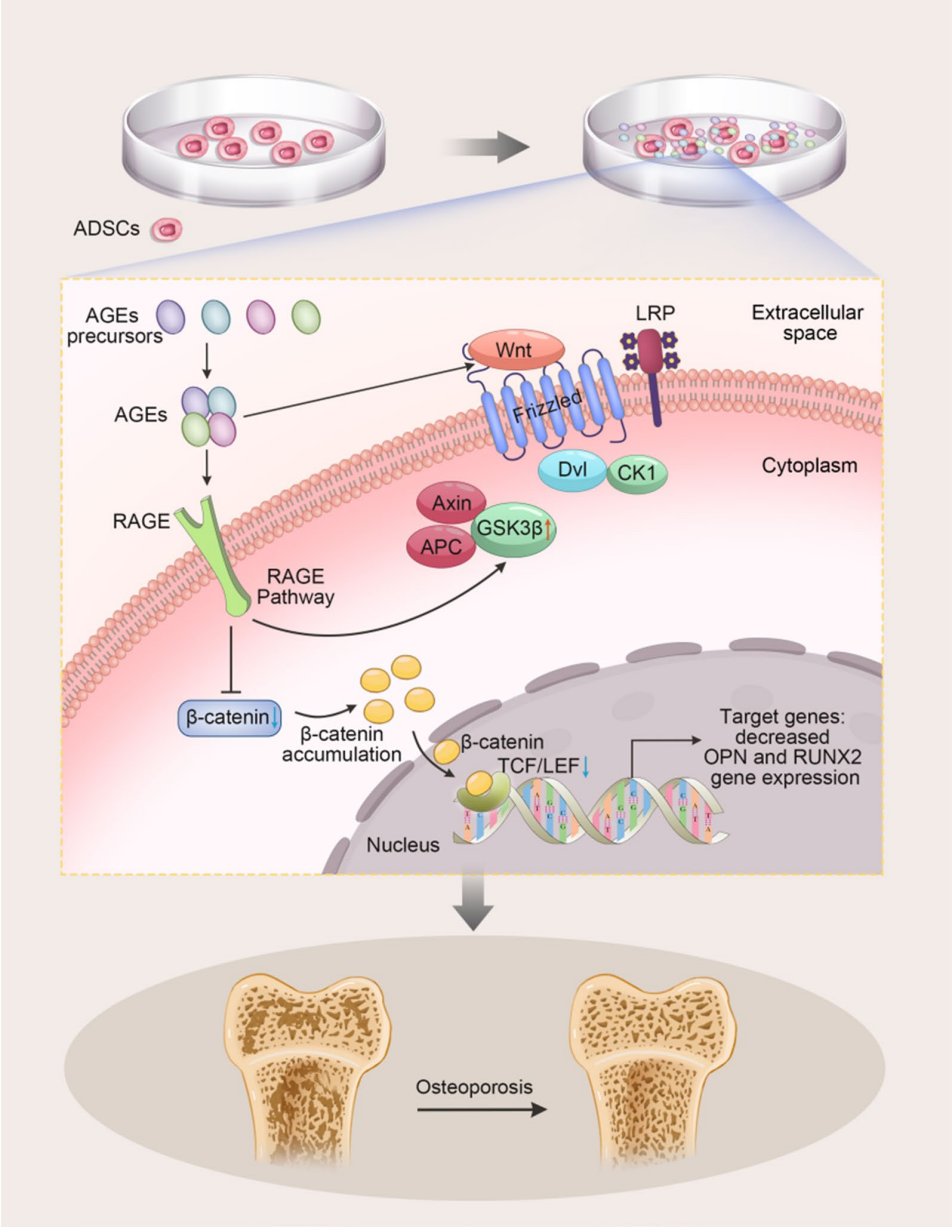
AGEs attenuate osteogenic differentiation ability of ADSCs by inhibiting Wnt/β-catenin pathway (Zhang et al., 2018; Li et al., 2020). In ADSCs, Wnt ligands interact with Frizzled and activate the Wnt signaling pathway, while AGEs inhibit the Wnt/β-catenin signaling pathway, which leads to an increase in phosphorylated β-catenin expression. β-catenin translocates into the nucleus and binds to TCF/LEF, leading to a decrease in LEF expression and inducing a decrease in OPN and RUNX2 expression

Different signaling pathways can regulate the multilineage differentiation potential of stem cells. Herein, we found two studies [38, 39] reporting on the role of the Wnt signaling pathway in stem cell differentiation. Li et al*.* [39] and Zhang et al*.* [38] found that AGEs decrease the osteogenic differentiation ability of ADSCs by activating the canonical Wnt/β-catenin pathway. It can be seen that the Wnt signaling pathway has an important role in the process of bone regeneration and osteoblast differentiation. In ADSCs, Wnt ligands interact with Frizzled and activate the Wnt signaling pathway, while AGEs inhibit the Wnt/β-catenin signaling pathway, which leads to an increase in phosphorylated β-catenin expression. β-catenin translocates into the nucleus and binds to TCF/LEF, leading to a decrease in LEF expression and inducing a decrease in OPN and RUNX2 expression(Fig. 2).

Previous studies demonstrated the adverse effects of AGEs on the osteogenic potential of ADSCs [91]. Xiao et al. [41] found increased adipose differentiation potential and decreased osteogenic differentiation ability of ADSCs in response to AGEs; in addition, during this process, hypoxia-induced miR-1248 decreased, an effect associated with the miR-1248/CITED2/HIF-1α pathway. HIF-1α has been reported to inhibit and enhance osteogenic, adipogenic, and tenogenic differentiation of ADSC [92–94]. HIF-1 is divided into two subunits, HIF-1α and HIF-1β, and HIF-1α mainly determines the activity of HIF-1. Moreover, HIF-1α under hypoxia has an important role in the differentiation potential of ADSCs [95]. Yu et al*.* [96] showed that indirect co-culture of ADSCs with tenocytes increased the differentiation of ADSCs into tenocytes, and hypoxia further enhanced the ability of ADSCs to differentiate into tenoblasts, accompanied by an increase in HIF-1α, and the use of HIF-1α inhibitors attenuated the effect of hypoxia on the differentiation of ADSCs. A hypoxic environment adversely affects ADSCs, but HIF-1α signaling promotes the differentiation of stem cells into tendons [97]. HIF-1α contributes to stem cell adaptation to hypoxic conditions and has an important role in cellular response regulatory mechanisms. Thus, the HIF-1α signaling pathway has an important role in the differentiation process of ADSCs.

Similar to the study by Guo et al*.* [25], Li et al*.* [42] found that AGEs led to the decreased osteogenic potential of SIRT3-associated ADSCs. SIRT3 is mainly located in mitochondria and has an important role in mitochondrial function and cellular homeostasis. Some studies suggest that mitotic abnormalities are closely associated with the dysfunction of bone marrow stem cells [98]. In addition, increasing evidence suggests that SIRT3 is associated with bone metabolic processes [99]. One study found that knockdown of SIRT3 resulted in dysregulation of mitochondrial homeostasis and decreased osteogenic differentiation potential [100], and in addition, knockdown of SIRT3 resulted in increased osteoclast activity, significantly increased bone resorption, and significant loss of bone mass [101]. Abnormal SIRT3 expression can lead to osteoporosis [25].

### Mechanisms of AGEs affecting NSCs differentiation

**Fig. 3:**
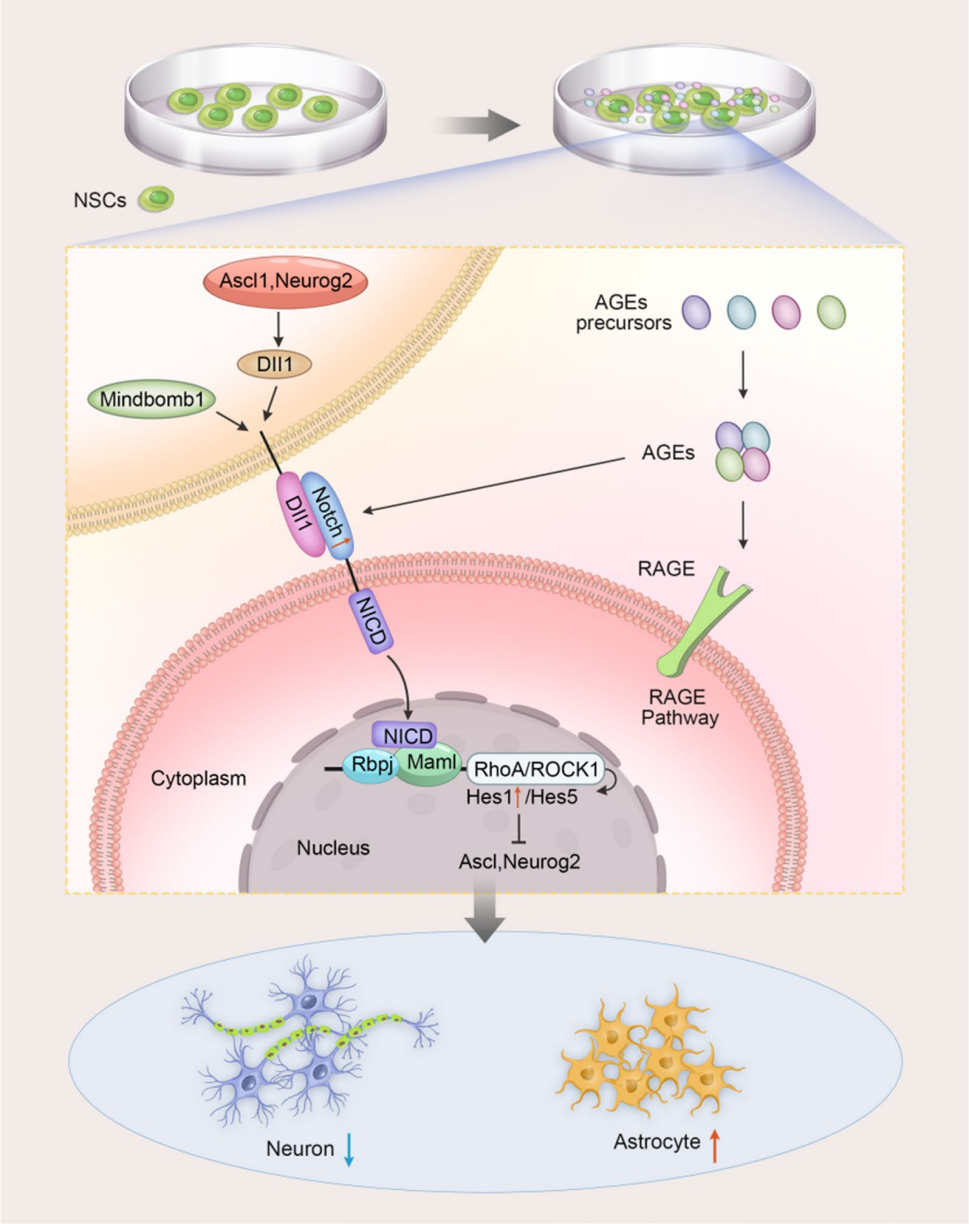
AGEs attenuated differentiation of NSCs into neurons and promoted differentiation into astrocytes by up-regulating Notch-Hes1 signaling (Guo et al., 2014). In NSCs, AGEs up-regulated Notch expression, and Notch signaling was activated by ligands that bind to Notch receptors, thereby triggering the release of the receptor intracellular domain (NICD), which then translocates to the nucleus and cooperates with the DNA-binding protein RBPJ and the transcriptional cooperative effector MAML to activate RhoA/ROCK expression. RhoA/Rock induces a significant increase in the expression of Hes1 and Hes5, especially Hes1, which in turn inhibits the expression of differentiation factors such as Ascl1 and Neurog2, ultimately leading to a decrease in the ability of NSCs to differentiate into neurons and promote the differentiation into astrocytes

Neurospheres can self-renew and differentiate into specific neurons, glial cells, and oligodendrocytes [102, 103]. Guo et al*.* [45] performed in vivo studies and found that AGEs can reduce the differentiation of NSCs into neurons and increase their differentiation into astrocytes by down-regulating the expression of the Notch-Hes1 signaling pathway. In NSCs, AGEs up-regulated Notch expression, and Notch signaling was activated by ligands that bind to Notch receptors, thereby triggering the release of the receptor intracellular domain (NICD), which then translocates to the nucleus and cooperates with the DNA-binding protein RBPJ and the transcriptional cooperative effector MAML to activate RhoA/ROCK expression. RhoA/Rock induces a significant increase in the expression of Hes1 and Hes5, especially Hes1, which in turn inhibits the expression of differentiation factors such as Ascl1 and Neurog2, ultimately leading to a decrease in the ability of NSCs to differentiate into neurons and promote the differentiation into astrocytes (Fig. 3).

It has been confirmed that Notch1-mediated pathways are involved in hippocampal neurogenesis under both physiological [104] and pathological conditions [105]. The Notch signaling pathway has an important regulatory role during embryonic development and acquired growth and development. Moreover, in mammals, the Notch signaling pathway has four Notch receptors, such as Notch1, and five Notch ligands, such as Jagged1 [106]. After the receptor binds to the ligand on the membrane, it is cleaved by gamma-secretase to release the Notch intracellular domain (NICD) into the nucleus and form a transcriptional activation complex after binding to the corresponding transcription factors, thereby regulating the expression of downstream target genes such as hairy division-related enhancers, including the *Hes1* gene [107, 108]. In addition, this pathway is required to maintain and expand the neural stem cell repertoire [109], and in regulating neural stem cell differentiation, it inhibits neural stem cell differentiation into neurons and promotes differentiation into glial cells [110, 111]. In sum, the Notch-Hes1 pathway is an important regulatory mechanism through which AGEs inhibit neurogenesis and promote astrocyte differentiation, providing potential therapeutic targets for hyperglycemia-related cognitive deficits.

Bao et al*.* [44] found that the expression level of Histone deacetylase 3 (HDAC3) was up-regulated in cultured NSCs after AGEs induction. AGEs inhibited neuronal differentiation and reduced neuronal regeneration in NSCs, and down-regulation of HDAC3 expression partially reduced the inhibitory effect of AGEs on neural stem cell differentiation. HDAC3 is one of the four members of human class I HDACs that has an important role in the proliferation and differentiation of neural stem cells [112]. HDAC inhibitors can induce neuron-directed differentiation (NPs) into neurons by directly up-regulating the expression of neuron-specific genes (NeuroD, Ngn1, and Math1) [113, 114] or exerting a regulatory role by activating the Notch/Hes signaling pathway [115]. In a study of differentiation of mouse NSCs, down-regulation of HDAC3 expression increased neuronal differentiation of NSCs [116], which is consistent with Bao’s findings [44]. To sum up, the above data suggest that AGEs inhibit the neuronal differentiation of NSCs by up-regulating the expression of HDAC3, while its potential molecular mechanism remains unclear. Future studies should focus on how HDAC3 regulates the differentiation of NSCs, and find key genes regulated by HDAC3 to explore the route for directionally inducing stem cells to differentiate into specific cells.

### Mechanisms of AGEs affecting EPCs differentiation

Wang et al*.* [48] showed that AGEs/RAGE promotes osteogenic differentiation of rat bone marrow EPCs through the MAPK signaling pathway. Mitogen-activated protein kinases (MAPK) signaling pathway, a group of mitogen-activated protein kinases that extracellular stimuli can activate, is an important carrier protein that transmits stimuli on the cell surface to the nucleus, including three kinases, including p38 mitogen-activated protein kinases (p38 MAPK), extracellular regulated protein kinases1/2 (ERK1/2), and c-Jun amino-terminal kinase (JNK), which are important components of intracellular signaling pathway transduction involved in a series of cell activities such as regulating cell proliferation, apoptosis, differentiation, and survival as well as functional synchronization between cells [117]. Notably, MAPK has a role in differentiating BMSCs [118], and osteoblast-specific gene expression is regulated by the MAPK pathway [119, 120]. In addition, the MAPK pathway activates osteopontin expression further to down-regulate osteogenesis and mineralization formation [121]. In the MAPK signaling pathway, the p38 pathway has an important role in cells' growth, survival, and differentiation, and regulation of p38 can promote the osteogenic differentiation of BMSCs [122]. Moreover, the ERK pathway, as the most classical pathway, mainly regulates the initial proliferation and differentiation of cells and plays an important role in osteoblasts [123], while the JNK pathway affects the activity of osteoblasts [124]. In summary, the MAPK signaling pathway has an important role in stem cell differentiation and is also an important regulatory mechanism through which AGEs affect the decrease in osteogenic differentiation of endothelial cells.

## Strategies to improve the differentiation capacity of primary stem cells by solving the problem of AGEs

The detrimental effects of AGEs on the differentiation of primary stem cells are often overlooked. Therefore, effective interventions are essential to promote stem cell differentiation in favorable directions. Combined with previous studies, we found that interventions tend to rely on blocking pathways in which AGEs act to reverse the detrimental effects of AGEs on stem cell differentiation; related mechanisms mainly include AGE/RAGE [24, 37, 38] and Wnt/β-catenin signaling pathways [33, 35, 38].

Blocking AGE/RAGE interaction is an effective strategy to reverse the adverse effects of AGEs on primary stem cells [31, 32]. Numerous studies have shown that AGE/RAGE interaction induces osteoblast apoptosis, reduces bone mass, and promotes osteoporosis in diabetic patients [125–127]. Zhang et al*.* [38] reported that FPS-ZM1, a RAGE inhibitor, could rescue the negative impact of AGEs on the osteogenic potential of ADSCs. Also, the authors found that FPS-ZM1 treatment resulted in decreased RAGE protein and mRNA. Twenty-one days later, the experimental results confirmed that alizarin red-S staining was significantly increased in PDLSCs treated with FPS-ZM1, while both OPN and Runx2 mRNA levels were increased. In previous studies, chondrooligomeric matrix protein angiopoietin 1 (COMP-Ang1) was demonstrated to promote osteoblast differentiation and bone formation [128–130]. Kim et al*.* [24] further confirmed this idea. COMP-Ang1 is thought to promote the enhancement of the osteogenic differentiation ability of BMSCs by affecting the p38/MAPK pathway and attenuating the expression of RAGE. Western blot (WB) results showed that COMP-Ang 1 significantly decreased the increase in RAGE expression induced by AGE treatment, and these results suggest that COMP-Ang1 may reverse the adverse effects of AGEs on BMSCs differentiation in part by decreasing the expression of RAGE; Wang et al*.* [37] found that periostin attenuated AGE-induced osteogenic inhibition of periodontal ligament stem cells by decreasing RAGE levels.

Abnormal changes in the Wnt signaling pathway are closely associated with bone metabolism [131, 132]. Some studies have shown that activation of the Wnt/β-catenin pathway promotes osteogenic differentiation of BMSCs, and treatment with high concentrations of WNT3a inhibits osteogenic differentiation of BMSCs [133–135]. Dickkopf-1 (DKK 1) can reverse the adverse effects of AGEs on PDLSCs through the mediated canonical Wnt/β-catenin pathway [33]. Also, DKK 1 can increase RUNX2 expression by inhibiting active β-catenin in PDLSC. Furthermore, β-catenin knockdown promotes osteogenic differentiation of PDLSC. Zhang et al*.* [35] found that AGEs activate the canonical Wnt/β-catenin signaling pathway and promote the nuclear translocation of β-catenin, while berberine partially rescues the AGEs-induced reduction in osteogenic potential of PDLSCs by inhibiting the canonical Wnt/β-catenin pathway. In addition, FPS-ZM1 has an important role in attenuating high glucose-induced BMSC inflammation [136]. Zhang et al*.* [38] reported that FPS-ZM1, a RAGE inhibitor, up-regulated the osteogenic potential of ASCs by partially regulating Wnt signaling.

At the same time, other studies have also provided strategies to reduce the adverse effects of AGEs on stem cells; however, these strategies are relatively independent. Wang et al*.* [34] found that GLP-1 may attenuate/inhibit the effect of AGEs in hPDLSC by inhibiting PKCβ2 phosphorylation, resulting in the increase in osteogenic genes and the enhancement of cell mineralization ability. GLP-1 (glucagon-like peptide-1), a 30/31-amino acid hormone, is an important modulator of bone growth and remodeling [23]. In addition, GLP-1 is important in reducing insulin resistance and promoting insulin secretion (139). Previous studies have found that GLP-1 receptor agonists facilitate the increase in bone mass and osteogenesis (140) and are effective in preventing the development of osteoporosis. It is believed that further research and application of GLP-1 in the prevention and treatment of diabetic osteoporosis are expected. Sirt3 has an important role in maintaining mitochondrial homeostasis, cellular energy supply, and biosynthesis, while cellular homeostasis is important for cellular differentiation [25, 101]. Abnormal expression of Sirt3 is closely related to bone metabolism disorders, so silencing Sirt3 expression can effectively prevent AGEs-induced osteoporosis [25]. Other studies [42] have also demonstrated that irisin decreases AGE-induced differentiation dysfunction in ADSCs by mediating Sirt3 expression. Thus, SIRT3-mediated intracellular mechanisms could serve as novel therapeutic strategies for bone regeneration under diabetic conditions in the future. Through the above interventions, the differentiation ability of stem cells can still be improved even if they are affected by AGEs, and these methods open up new therapeutic ideas for regulating the differentiation ability of primary stem cells.

## Conclusion

This systematic review summarizes the effects of AGEs on the differentiation potential of various types of primary stem cells and how this abnormal differentiation of primary stem cells affects the body. AGE/RAGE and Wnt/β-catenin signaling pathways are considered important regulatory mechanisms through which AGEs affect the differentiation ability of primary stem cells. In the future, more effective approaches are needed to address the negative impact of AGEs on the differentiation properties of primary stem cells.

## Data Availability

Not applicable.

## Abbreviations

AGEs: Advanced glycation end products
ADSCs: Adipose tissue-derived stem cells
BMSCs: Bone marrow stem cells
MSCs: Marrow stem cells
PDLSCs: Periodontal ligament stem cells
NSCs: Neural stem cells
TDSCs: Tendon stem cells
EPCs: Endothelial progenitor cell
SC: Stem cell
ALP: Alkaline phosphatase
NICD: Notch intracellular domain
HDAC3: Histone deacetylase 3
PKC: Protein kinase C
ROS: Reactive oxygen species
COMP-Ang1: Cartilage oligomeric matrix protein angiopoietin 1
WB: Western blot
OPN: Osteopontin
MAPK: Mitogen-activated protein kinases
ERK1/2: Extracellular regulated protein kinases1/2
JNK: C-Jun amino-terminal kinase
TGF-β: Transforming growth factor-beta
